# Molecular Mechanisms behind Free Radical Scavengers Function against Oxidative Stress

**DOI:** 10.3390/antiox6030051

**Published:** 2017-07-10

**Authors:** Fereshteh Ahmadinejad, Simon Geir Møller, Morteza Hashemzadeh-Chaleshtori, Gholamreza Bidkhori, Mohammad-Saeid Jami

**Affiliations:** 1 Cellular and Molecular Research Center, Shahrekord University of Medical Science, Shahrekord 88157, Iran; Fereshte.ahmadi86@ymail.com (F.A.); mchalesh@yahoo.com (M.H.-C.); 2 Department of Biological Sciences, St John’s University, New York, NY 11439, USA; 3 Norwegian Center for Movement Disorders, Stavanger University Hospital, Stavanger 4068, Norway; 4 SciLifeLab, KTH Royal Institute of Technology, Solna 17165, Sweden; gholamreza.bidkhori@scilifelab.se

**Keywords:** neurodegenerative disease, oxidative stress, L-DOPA, Edaravone, proteomics

## Abstract

Accumulating evidence shows that oxidative stress is involved in a wide variety of human diseases: rheumatoid arthritis, Alzheimer’s disease, Parkinson’s disease, cancers, etc. Here, we discuss the significance of oxidative conditions in different disease, with the focus on neurodegenerative disease including Parkinson’s disease, which is mainly caused by oxidative stress. Reactive oxygen and nitrogen species (ROS and RNS, respectively), collectively known as RONS, are produced by cellular enzymes such as myeloperoxidase, NADPH-oxidase (nicotinamide adenine dinucleotide phosphate-oxidase) and nitric oxide synthase (NOS). Natural antioxidant systems are categorized into enzymatic and non-enzymatic antioxidant groups. The former includes a number of enzymes such as catalase and glutathione peroxidase, while the latter contains a number of antioxidants acquired from dietary sources including vitamin C, carotenoids, flavonoids and polyphenols. There are also scavengers used for therapeutic purposes, such as 3,4-dihydroxyphenylalanine (L-DOPA) used routinely in the treatment of Parkinson’s disease (not as a free radical scavenger), and 3-methyl-1-phenyl-2-pyrazolin-5-one (Edaravone) that acts as a free radical detoxifier frequently used in acute ischemic stroke. The cell surviving properties of L-DOPA and Edaravone against oxidative stress conditions rely on the alteration of a number of stress proteins such as Annexin A1, Peroxiredoxin-6 and PARK7/DJ-1 (Parkinson disease protein 7, also known as Protein deglycase DJ-1). Although they share the targets in reversing the cytotoxic effects of H_2_O_2_, they seem to have distinct mechanism of function. Exposure to L-DOPA may result in hypoxia condition and further induction of ORP150 (150-kDa oxygen-regulated protein) with its concomitant cytoprotective effects but Edaravone seems to protect cells via direct induction of Peroxiredoxin-2 and inhibition of apoptosis.

## 1. Introduction

The term oxidative stress is commonly used to describe an imbalance between the systemic manifestation of free radicals and the capability of cells to detoxify them and negate their damaging effects on proteins, lipids, and DNA [1]. The perceptual origin of “oxidative stress” is tracked back to the 1950s and the term began to be used frequently by scientists from 1970 as they started to unravel the effects of free radicals and ionizing radiation [2]. The important relationship between oxidative stress and a wide variety of human diseases has placed this stress factor at the forefront of diseases research. Indeed, diseases such as rheumatoid arthritis (RA) [3,4], Alzheimer’s disease (AD), Parkinson’s disease (PD), amyotrophic lateral sclerosis (ALS) [5], cardiovascular disease [6], allergies [7], immune system dysfunctions [8], diabetes, and cancer are all related to oxidative stress.

The important intracellular signaling molecules in RA are reactive oxygen species (ROS), which may damage matrix components and enhance the synovial inflammatory proliferative response in immune system cells [9]. Oxidative stress conditions may also make T-cells resistant to growth or death stimulators [10]. Moreover, the pathological role of mitochondrial respiratory chain dysfunction and also the roles of oxidative stress in neurodegenerative disease such as AD and PD are well known. The identification of mutations in a few specific genes involved in PD indicates the relevance of both mitochondrial dysfunction and oxidative stress in the sporadic and familial forms of the disease. All of the proteins associated with familial forms of PD are involved in the pathways of oxidative stress and free radical damage. These proteins, including PINK1 (PTEN-induced putative kinase 1), DJ-1 (Parkinson disease protein 7, also known as Protein deglycase DJ-1), LRRK-2 (Leucine-rich repeat kinase 2), parkin and α-synuclein (SNCA), are associated with mitochondria or are mitochondrial proteins [11]. The difference between the prevalence of sporadic and familial forms of the disease may provide a hint to address the question to what extent the mitochondrial injury in PD is due to genetic origin and how much is caused by hydrogen peroxide generated during enhanced turnover of dopamine neurons. However, despite this knowledge, further investigation is needed to reveal the detailed molecular etiology of PD.

Additionally, the increase of cellular ROS is strongly linked to LDL (low-density lipoprotein) oxidation, endothelial dysfunction and other pathological conditions in cardiovascular diseases [12,13]. Various studies have discussed the role of oxidative stress and increased levels of hydrogen peroxide [14,15,16] and nitric oxide [17] in allergic diseases such as asthma. Insulin resistance and enzymatic dysfunctions are oxidative stress effects in diabetes resulting in glucose oxidation and increased lipid peroxidation [18]. Among the complex diseases, cancer initiation and progression is mediated by many different predisposing factors including the activation of different proto-oncogenes [19] and alteration of microRNAs [20,21,22]. The role of ROS and oxidative stress has been investigated extensively in cancer. It is known that the activation of proto-oncogenes in carcinogenesis may be a result of the formation of the highly reactive hydroxyl radical (OH•) [23].

Since oxidative stress affects a broad spectrum of cells, organelles, and molecular pathways, new insight into these various processes will contribute to the development of new therapeutic strategies, especially for neurodegenerative disease. In this study, we focus on the role of oxidative stress as well as mechanisms of scavengers function in neurodegenerative disease. We have included some recent finding provided by proteomic studies [24,25,26,27].

## 2. Neurodegenerative Disease

The most common chronic neurodegenerative diseases are AD and PD. The AD is the most common type of dementia, accounting for 60–70% of dementia cases [28]. The AD has a progressive pattern defined by short-term memory loss at first and overall cognitive functioning and behavioral symptoms particularly during advanced stages. The two main neuropathological characteristics of AD are abnormal deposition of amyloid β (Aβ) peptide and intracellular accumulation of neurofibrillary tangles of hyperphosphorylated τ protein [29]. Inflammatory processes may also play an important role in the pathogenesis of AD [30]. According to published data, oxidative stress increases in the aging brain leading to increased levels of radical species which ultimately disrupts the balance of antioxidant systems where the expression of stress response, antioxidant and DNA damage repair genes decreases [31]. Although sufficient evidence indicates that oxidative stress is involved in the cellular damage that leads to neuropathology, it remains obscure whether free radical-induced oxidative stress is the primary, initiating event causing neurodegeneration or a downstream consequence of tissue injury.

Moreover, the balance between mitochondrial fusion and fission and the dynamic movement of mitochondria is disrupted with age. It is well known that neurons are highly dependent on appropriate mitochondrial functions because of their high requirement of energy. In addition to the regulation of apoptosis process, studies on mitochondrial dysfunction conditions have outlined the role of this organelle in ROS production in synaptic areas and intracellular spaces [32].

The second most prevalent neurodegenerative disorder is PD [32]. PD mainly affect motor functions in the central nervous system and is characterized by the progressive death of dopaminergic neurons within the substantia nigra and the accumulation and aggregation of the alpha-synuclein (SNCA) protein [33]. In the early stages of PD, the most common symptoms include tremors, muscle rigidity, postural instability and gait [34]. In later stages, non-motor behavioral symptoms including dementia, depression, and insomnia may arise [35]. Some of the molecular mechanisms associated with the pathogenesis of PD have been identified and several proteins have been directly linked to the disease. These include SNCA [36], LRRK2 [37], PARK7/DJ-1 [38], PINK1 [39], Lewy body-positive PARK1/parkin [39] and Lewy body-negative juvenile PARK2 [40]. A number of cellular mechanisms have also been unraveled including defective protein folding, mitochondrial dysfunction, inflammation and oxidative stress [41]. In PD, free radicals may be produced and accumulated in neurons through several mechanisms. These mechanisms include lipid peroxidation [42], glutathione deficiency [43], iron accumulation [42], ferritin reduction [44], and defective mitochondrial respiratory chain functions [42].

## 3. Sources of Oxidative Stress

Oxidative stress can be broadly divided into exogenous and endogenous mechanisms. Although very different in origin, excessive oxidative stress may lead to damage to lipids, proteins and DNA. Conversely, oxygen and nitrogen free radicals are essential for all aerobic organisms where free radicals and ROS are used for advantageous biological effects. The regulation of free radicals in maintaining redox homeostasis is therefore essential for the physiological health of organisms.

Exogenous and environmental sources of oxidative stress include ionizing radiation of X-, γ- or cosmic rays and α particles from radon decay. Furthermore, many exogenous factors may interact with different classes of chemicals leading to a variety of stress insults ultimately [45,46]. The other types of environmental sources for oxidative stress could be enumerated as pesticides and environmental chemicals. The toxicity effect of pesticides is mainly due to their ability to generate free radicals and subsequently peroxidation of biomolecule and alteration of scavenger enzymes [47]. The role of some solvents has also been clarified in ROS generation and oxidative stress conditions. For example the in vitro effects of six solvents (*n*-hexane, *n*-octane, toluene, *n*-butyl benzene and cyclohexane) have been assessed on rat cerebellum granular cell cultures and showed some levels of ROS generation [48]. Additionally, the ability of redox-active metals such as iron (Fe), copper (Cu), chromium (Cr), and cobalt (Co)) in generation of superoxide anion radical and nitric oxide radicals have been widely studies. For example, it is known that excessive amounts of Iron and copper can be toxic for cells as they affect the redox state of the cell [49].

Endogenous stress often has an intracellular origin mainly derived from signaling pathways, and metabolic and/or inflammation processes [50]. Moreover, some studies indicate the role of cultural condition of cells in changing the expression patterns of different genes and their DNA stability through oxidative stress mechanisms [51]. Indeed, metabolic processes may produce different types of ROS, including superoxide (O_2_•^−^), hydrogen peroxide (H_2_O_2_), hydroxyl radicals (OH•) and singlet oxygen (O_2_), which can, if present at an inappropriate level, oxidize DNA and induce various types of damages such as double-stranded DNA breaks and impairments which are frequently found in human tumors [50]. In addition to ROS-producing enzymatic processes which involve NADPH oxidase, xanthine oxidase, uncoupled endothelial nitric oxide synthase (eNOS), cytochrome P450 enzymes, lipoxygenase, and cyclooxygenase, there are non-enzymatic reactions such as mitochondrial respiratory chain [52,53]. The natural production of ROS by the mitochondrial respiratory chain is complex as the ROS can be beneficial in metabolic standpoint but at the same time detrimental to cells [54,55]. It is noteworthy that the effects of free radicals are concentration dependent allowing ROS to toggle between beneficial and harmful scenarios in a pseudo yin and yang fashion. One of the beneficial roles of oxidative stress includes the defense against infections and pathogens. Cells of the immune system, especially neutrophils, undergo a series of reactions in confronting foreign particles by triggering a process called respiratory burst. In this process, phagocytic cells, such as macrophages and neutrophils, produce a massive amount of ROS and superoxide radicals. NAD(P)H oxidase (nicotinamide adenine dinucleotide phosphate oxidase) is the best known enzyme in phagocytic cells that produces O_2_•^−^ for bacterial destruction within the respiratory burst process [56]. It has also been demonstrated that ROS can act as secondary messengers in intracellular signaling pathways acting as anti-tumorigenic factors by inducing cell senescence and apoptosis [57]. Conversely, low levels of H_2_O_2_ may act as a signaling molecule, promoting cell proliferation, differentiation and migration [58]. Combined, it is clear that ROS biology is complex where cells have evolved intricate mechanisms to modulate ROS levels, ensuring redox homeostasis, depending on cell state and fate.

## 4. RONS (Reactive Oxygen and Reactive Nitrogen Species)

ROS and reactive nitrogen species (RNS) are collectively called RONS. These types of free radicals are mainly produced by cellular enzymes such as NADPH-oxidase, myeloperoxidase and nitric oxide synthase (NOS).

Reactive nitrogen species (RNS) act with reactive oxygen species (ROS) to induce nitrosative stress condition. In animal cells, RNS production starts with the reaction of superoxide (O_2_•^−^) with nitric oxide (NO•) to form peroxynitrite (ONOO^−^), which is a highly reactive species with the ability to damage lipids, DNA bases, proteins, thiols, etc. Since generation of RNS is linked to ROS, it is not surprising that scavengers and antioxidants could reduce the formation and activity of RNS and thus nitrosative stress condition.

The evolutionary role of low concentrations of RONS is known in the immune system where these radicals will eliminate foreign entities within cells to benefit the organism [59]. A growing body of evidence suggests an important role of RONS in intracellular signaling pathways and in processes such as regulating vascular tone, insulin synthesis, hypoxia-inducible factor (HIF) activation, cell proliferation, differentiation and migration [60,61]. In contrast, excessive levels of these free radicals may cause harmful effects on biological structures as discussed previously [62]. Indeed, one of the most susceptible RONS targets is the proteome, the vital machinery for cellular protein homeostasis.

Despite the harmful effects of RONS, a number of proteins are resistant to oxidative stress because of ROS-mediated thiol modifications particularly in cysteine residues. The thiol modification is involved in the regulation of protein function and structure under stress conditions [63]. Depending on the cell’s redox-state, the thiol group of the cysteine residue can be either reduced to a free thiol (SH) by the antioxidant defense system or converted to other oxidative post-translational modifications (Ox-PTMs) [64].

Protein oxidation has been well shown in brains undergoing neurodegeneration. For example, protein carbonyls [65,66] and 3-nitrotyrosine [67] are observed in Alzheimer’s disease (AD) and Parkinson’s disease (PD) brains [68]. Indeed, proteomics approaches in AD brains have revealed a number of oxidized proteins including creatine kinase BB (CK), glutamine synthase (GS), ubiquitin carboxy-terminal hydrolase L-1 (UCHL1), α-enolase, and dihydropyrimidinase related protein 2 (DRP2) [66,69]. Creatine kinase (BB isoform), α-enolase, and triosephosphate isomerase are involved in energy production processes in cells and it seems that a reduction in ATP (Adenosine triphosphate) levels may initiate the degeneration of neurons in AD brain [70]. Proteomic studies further revealed that α-synuclein, a hallmark protein of PD (which lacks tryptophan and cysteine residues), undergoes methionine oxidation to form methionine sulfoxides in the substantia nigra in PD brains. Methionine oxidation has inhibitory effects on protein fibrillation. This can result in the subsequent α-synuclein aggregation, which in turn may influence the onset and progression of PD [71].

## 5. Scavengers

The study of scavengers and their beneficial role within imbalanced redox situations may provide new insights into new therapeutic strategies for oxidative stress-related diseases. Natural antioxidant systems are categorized into enzymatic and non-enzymatic antioxidant groups. The enzymatic group include a number of enzymes such as catalase, the enzymes of glutathione thioredoxin system and superoxide dismutase (SOD) [72]. Catalases exist in eukaryotic peroxisomes and catalyze the conversion of H_2_O_2_ into water and oxygen in the presence of iron or manganese cofactors [73]. The glutathione system comprises of three enzymes, glutathione reductase, glutathione peroxidases and glutathione S-transferases, that contribute in breakdown of H_2_O_2_ and hydroperoxides using selenium as the cofactor [74,75]. The thioredoxin system comprises of thioredoxin protein and thioredoxin reductase and acts as a scavenging factor for ROS, a phenomenon that makes other proteins stable in a reduced state [76]. Superoxide dismutases are also antioxidant enzymes that work with cooper/zinc in the cytosol and manganese in mitochondria catalyzing the breakdown of superoxide anions into oxygen and H_2_O_2_ [77,78].

The non-enzymatic group contains a number of antioxidants that act directly on oxidative agents and are acquired from dietary sources. This group includes vitamin C (Ascorbate), vitamin E (-tocopherol), carotenoids, flavonoids, polyphenols etc. Ascorbate plays an important role in detoxification of peroxyl and hydroxyl radicals, superoxide, singlet oxygen, and peroxynitrite in many organs particularly in the brain [79]. Vitamin E can interact with ascorbate and protect brain cells during stress conditions [80,81].

There are also antioxidants that are produced by cells and chelate and/or bind to redox metals thus protect the cells against oxidative stress indirectly [82]. Melatonin, one of the first examples of natural scavengers is known to be involved in neutralization of hypochlorous acid and also detoxify a wide range of environmental and chemical agents including H_2_O_2_, hydroxyl radical (OH•), peroxyl radicals (ROO•), singlet oxygen (^1^O_2_), and reactive nitrogen species (RNS) such as nitric oxide radical (NO•) and peroxinitrite (ONOO^−^) [83,84,85]. There are several mechanism in which melatonine is involved in oxidative stress protection including electron donation, hydrogen donation, addition, substitution, nitrosation and repair [86]. Other endogenously produced antioxidants are ubiquinol (coenzyme Q), a product of the mevalonate pathway [87] and glutathione synthesized from the amino acids L-cysteine, L-glutamic acid, and glycine.

Some scavengers such as 3,4-dihydroxyphenylalanine (L-DOPA) are currently used for therapeutic purposes for PD treatment [88,89]. Although widely used as a symptomatic relief therapeutic for PD there are conflicting data on its negative effect on dopaminergic neurons and its positive effects on motor system [90]. Another scavenger is Edaravone (3-methyl-1-phenyl-2-pyrazolin-5-one) that acts as a free radical detoxifier frequently used in acute ischemic stroke [91]. The scavenging role of Edaravone against oxygen and hydroxyl radicals, and its inhibitory role in lipid peroxidation/lipooxygenase pathways, suggests that Edaravone may be a potential therapeutic for oxidative-induced diseases [92,93,94,95].

## 6. L-DOPA

L-DOPA can activate different mechanisms in central neurons most of which triggered by the transformation of L-DOPA to dopamine (DA) and noradrenalin (NA). All of the D1 and D2 types of DA receptors are activated by DA produced from L-DOPA through both direct and un-conventional receptors. NA, derived from L-DOPA, also helps NA-mediated activation of α- and β-adrenoceptors [96]. Under normal physiological conditions, DA is transported to synaptic terminals and stored until being used as neurotransmitter [97]. A number of neurological diseases are closely correlated with DA system dysfunction and the most common medications used for their treatment are based on altering the effects of DA. PD, Schizophrenia and attention deficit hyperactivity disorder (ADHD) are some of examples of these diseases [98,99]. Although the use of L-DOPA is widespread, controversial data exist on the beneficial versus harmful effects of this compound, particularly in the treatment of PD. For example, studies have shown that L-DOPA can damage DA neurons because of oxidative stress or other unknown mechanisms [90,100]. Further, Reksidler and co-workers showed that intraniagral injection of L-DOPA significantly reduces the number of DA neurons [101]. By contrast, there is much evidence that suggest that L-DOPA has neuroprotective effects by decreasing lipid peroxidation [27,102]. Additionally, studies have demonstrated that L-DOPA can protect neurons by neutralizing the effects of strong oxidants [103].

In an attempt clarify the mechanisms of L-DOPA in terms of neuroprotection, several proteomics studies have been conducted. One of these studies showed that L-DOPA could reverse the toxic effect of H_2_O_2_ on the morphology and viability of SHSY5Y human neuroblastoma dopaminergic cells, and also reduce ROS levels in response to H_2_O_2_ exposure [100]. According to this report, the expression of ten proteins were significantly up-regulated in H_2_O_2_ treated neuronal cells: Fumarate hydratase, Hydroxyacyl-coenzyme A dehydrogenase, Cofilin-2, Destrin, Cofilin-1, Nucleoside diphosphate kinase, L-lactate dehydrogenase A-like 6B, Annexin A1, Protein DJ-1 and Peroxiredoxin-6 [104]. On the other hand, significant down-regulation of six other proteins was observed in response to the H_2_O_2_-based oxidative condition. These proteins are: Vimentin, Tropomyosin alpha-4 chain, Tropomyosin alpha-1 chain, Glyceraldehyde-3-phosphate dehydrogenase, Cathepsin Z and Peptidyl-prolyl cis-trans isomerase D. Similarly, exposure to L-DOPA resulted in a significant increase in expression of Hypoxia up-regulated protein 1. Furthermore, the co-treatment condition (treatment with both L-DOPA and H_2_O_2_) was also exerted in the same study to examine the probable protective role of L-DOPA in excess oxidative stress. Although the oxidative stress affects cytoskeletal integrity and leads to various changes in neuronal metabolic routes L-DOPA appears to reverse the H_2_O_2_^−^ mediated effects. Particularly, the expression of Hypoxia up-regulated protein 1 was just observed in the presence of active L-DOPA [104]. This knowledge suggests that L-DOPA may participate in a cell survival mechanism which is triggered by deprivation of oxygen through induction of ORP150. In the pathway of catecholamine synthesis, the conversion of L-DOPA to norepinephrine through dopamine requires molecular oxygen. This indicates that L-DOPA can aid hypoxia condition and therefore induction of ORP150 with its concomitant cytoprotective effects [104].

Moreover, the expression of seven proteins, prolidase, actin-related protein 2, F-actin-capping protein subunit β, tropomyosin α-3 chain, proteasome activator complex subunit 1, peroxiredoxin 6, and a glyceraldehyde-3-phosphate dehydrogenase that are all involved in cellular integrity, were significantly down-regulated in patients that were administered with L-DOPA [105]. Furthermore, in a study conducted to identify protein deregulation in PD patients, a number of proteins showed different patterns of expression. It was shown by 2D-PAGE (Two Dimensional Polyacrylamide gel Electrophoresis) that the expression of three proteins, Cofilin-1, actin and mitochondrial ATP synthase β-subunit, were significantly up-regulated in patients whereas tropomyosin and γ-fibrinogen were down-regulated [106]. According to this report, there was no linear correlation between the protein profile and the L-DOPA dose [106]. In a separate study, it was shown that the expression of 60-kDa heat shock protein (HSP-60) was up-regulated in cells exposed to L-DOPA under oxidative conditions [107]. This protein is part of the Hsp60/procaspase-3 complex and plays a protective role in cells [108]. The other cytoprotective mechanism of L-DOPA occurs in hypoxia condition with up-regulation of hypoxia up-regulated protein 1 (ORP150), a protein folding chaperone [109]. Despite the broad spectrum of protein profile studies on neuronal cells in response to L-DOPA exposure, additional experiments are required to clarify the complex role of L-DOPA in either cytoprotective or cytotoxic mechanisms.

## 7. Edaravone

Edaravone (3-methyl-1-Phenyl-2-pyrazolin-5-one) is a free radical scavenger used for therapeutic purposes in neural damage. Edaravone has combined properties of vitamin C and vitamin D in inhibition of free-radical induced peroxidation [95,110,111]. The scavenging property of Edaravone has been proven in both lipid and aqueous phases as it works against free radicals, particularly hydroxyl radicals [111]. Lipid peroxidation process mainly begins by production of lipid radicals (^.^L) and since the lipid peroxidation process is depend on OH• generation, Edaravone has the capability in preventing this mechanism by scavenging the responsible radicals such as OH• [111]. The beneficial role of Edaravone has been investigated in a wide range of disease such as Parkinson’s disease, Alzheimer’s disease, atherosclerosis, chronic heart failure and diabetes mellitus [112,113,114,115,116]. To clarify the exact role of Edaravone, a number of proteomics studies have been conducted. For example, the protein profile of brain microvascular endothelial cells was investigated using two-dimensional fluorescence difference gel electrophoresis (2D-DIGE). The results revealed that the expression of five different groups of proteins altered upon Edaravone treatment. These groups are: Cytoskeleton (Vimentin, actin, POTE ankyrin domain family member F and Rho-related guanosine triphosphate-binding protein RhoH), the Glycometabolism related proteins (Pirin, fructose-bisphosphate aldolase A, pyruvate kinase isozymes M1/M2, and α-galactosidase A), the antioxidant group (NADPH: adrenodoxin oxidoreductase), translational proteins, and some other proteins [117]. In another study, the cytoprotective effects of Edaravone were investigated in SHSY5Y dopaminergic neurons [118]. Edaravone treatment was found capable to reverse the cytotoxic effects of H_2_O_2_ on viability and morphology of cells. Lactate dehydrogenase (LDH) Cytotoxicity and Terminal deoxynucleotidyl transferase (TdT) dUTP Nick-End Labeling (TUNEL) assays were also performed to study and compare the different impacts of H_2_O_2_ and Edaravone in treatment conditions. These data indicate that the apoptosis rate of treated cells is significantly elevated in presence of H_2_O_2_, however this effect is mostly reversed when co-treated with Edaravone. Further proteomics analysis of different treatment conditions showed that the expression of mitochondrial Fumarate hydratase, Hydroxyacyl coenzyme A dehydrogenase, Cofilin-2, Destrin, Cofilin-1, Nucleoside Diphosphate kinase, Peroxiredoxin-6, Vimentin, Tropomyosin alpha-4 chain, Tropomyosin alpha-1 chain, are significantly up-regulated in H_2_O_2_ stress condition. Conversely, down-regulated proteins included L-lactate Dehydrogenase A-like 6B, Annexin A1, Protein DJ-1, gelyceraldehyde-3-phosphate dehydrogenase, Cathepsin Z, Peptidyl-prolyl cis-trans isomerase D. The most interesting finding from the above mentioned study is the great up-regulation of Peroxiredoxin-2 and down-regulation of Protein disulfideisomerase A3 in response to Edaravone treatment [118]. Among the Peroxiredoxins (PRXs), Peroxiredoxins-2 (PRX2) levels are elevated in the substantia nigra of PD patientsm [68]. PRX2 is also known to protect neuronal cells against oxidative stress via attenuation of the apoptosis signal regulating kinase (ASK1) signaling cascade and therefore increase the cellular viability [119]. Overall consideration of the altered proteins after different treatments suggests that Edaravone scavenges H_2_O_2_ and protects cells against oxidative stress via direct induction of Peroxiredoxin-2 and inhibition of apoptosis [118].

## 8. Edaravone versus L-DOPA

As two cytoprotective reagents, L-DOPA and Edaravone are involved in different cytosolic and mitochondrial pathways to protect cell against oxidative stress. One of the common proteins through which L-DOPA or Edaravone engage to cell rescue is GAPDH (Glyceraldehyde-3-phosphate dehydrogenase), which plays an important role in Glycolysis pathway. This alteration potentially results in a metabolic flux change from glycolysis to the pentose phosphate pathway (PPP) allowing cells to produce more NADPH to prevent damages caused by oxidative stress. Indeed, NADPH is known to have protective effects in oxidative condition through conversion of the H_2_O_2_• to H_2_O. During oxidative stress the cellular machinery promptly changes accordingly, so that the glucose metabolism shifts from the glycolysis to pentose phosphate pathway to increase the generation of NADPH and moderate the oxidative effects [120,121]. As mentioned above, the expression levels of GAPDH protein is significantly down regulated in oxidative stress however treatment with either L-DOPA or Edaravone leads to compensation of GAPDH levels. In addition to the cytosolic mechanisms, mitochondrial routs are also altered in oxidative stress condition leading to lower levels of NAD(P)H generation but treatment with either L-DOPA or Edaravone reverses these effects [104,118].

The cell surviving property of L-DOPA and Edaravone against oxidative stress conditions relies on the up-regulation of a number of stress proteins such as Annexin A1, Peroxiredoxin-6 and PARK7/DJ-1. Annexin A1 is a membrane associated protein that plays an important role in NF-κB (nuclear factor kappa-light-chain-enhancer of activated B cells) signaling pathway for regulation of proliferation and apoptosis as a redox sensor [122,123]. The Peroxiredoxin-6 protein could also reduce the H_2_O_2_ and short chain fatty acid/phospholipid hydroperoxides and inhibit the NF-κB mediated death signaling [124,125]. PARK7/DJ-1 has been studied in mitochondria function, transcriptional regulation and oxidative conditions, and its cytoprotective effects against neural cell apoptosis by inhibiting p53-Bax-caspase pathway is evident [126,127]. Cathepsin X, a lysosomal cysteine proteinase that acts in cell death mechanisms, is known to be significantly down-regulated in oxidative conditions, but, similar to many other effects, this phenomenon is reversed by either L-DOPA or Edaravone. Cathepsin X targets a number of neurotropic proteins such as α and γ enolases, and mediates neuronal cell survival and neurogenesis [128]. Moreover, the viability and morphology of cells is really depended on cytoskeletal integrity which is vigorously affected in oxidative stress condition. Based on proteomics results, several structural proteins are significantly altered during oxidative stress and these effects are fully reversible by administration with either L-DOPA or Edaravone [104,118]. For instance, Cofilin-1, cofolin-2 and Destrin, which are actin depolymerizing factors and regulate cytoskeleton organization, together with other contractile proteins such as Tropomyosin alpha-4 chain, are known to be altered in oxidative stress conditions effects [104,118,129,130].

Although L-DOPA and Edaravone treatments share the targets in reversing the cytotoxic effects of H_2_O_2_, they seem to differ in mechanism of function. As mentioned above the exposure to L-DOPA could result in hypoxia condition in cells and this situation induces ORP150 that has cytoprotective effects [104]. However, it cannot be ruled out that L-DOPA itself has ROS scavenging function. Similarly, Edaravone scavenges H_2_O_2_, but it protects cells against oxidative stress via direct induction of Peroxiredoxin-2 and inhibition of apoptosis [118].

## 9. Conclusions

Oxidative damage is one of the main causes of neurodegenerative diseases such as Alzheimer’s disease, Huntington’s disease, Parkinson’s disease, and amyotrophic lateral sclerosis. Despite a wide variety of studies on oxidative stress phenomenon and role of scavengers, there are still various gaps in explanation of the exact mechanism of these scavengers function. The cytoprotective effects of L-DOPA and Edaravone are similar in affecting protein profile during treatment conditions but the exact mechanism of their function differs and needs further investigations. The L-DOPA function in central neurons is triggered by the transformation of L-DOPA to dopamine (DA) and noradrenalin (NA) and this process would be helpful for dopamine deficiency compensation and nerve signal transmission. Protein profile changes also suggest that L-DOPA could increase the level of cellular NAD(P)H levels through different pathways and decrease the level of free radicals in cell. The Edaravone function is less known, but according to the protein profile changes in treatment experiments, its role is closely similar to L-DOPA. The function of Edaravone is related to lipid peroxidation process, which mainly begins by production of lipid radicals (L•). Edaravone could prevent this mechanism by scavenging the responsible radicals such as OH•, because the lipid peroxidation process is dependent on OH• generation. Although the therapeutic use of Edaravone is wide spread in Eastern Asia, it has not been yet approved by FDA (Food and Drug Administration) and further investigations are definitely required to assess the benefits and safety.

## References

[1] Chandra K., Salman A.S., Mohd A., Sweety R., Ali K.N. (2015). Protection against FCA induced oxidative stress induced DNA damage as a model of arthritis and in vitro anti-arthritic potential of costus speciosus rhizome extract. Int. J. Pharmacogn. Phytochem. Res..

[2] Gerschman R., Gilbert D.L., Nye S.W., Dwyer P., Fenn W.O. (1954). Oxygen poisoning and X-irradiation: A mechanism in common. Science.

[3] Stamp L.K., Khalilova I., Tarr J.M., Senthilmohan R., Turner R., Haigh R.C., Winyard P.G., Kettle A.J. (2012). Myeloperoxidase and oxidative stress in rheumatoid arthritis. Rheumatology.

[4] Hassan S.Z., Gheita T.A., Kenawy S.A., Fahim A.T., EL-SOROUGY I.M., Abdou M.S. (2011). Oxidative stress in systemic lupus erythematosus and rheumatoid arthritis patients: Relationship to disease manifestations and activity. Int. J. Rheum. Dis..

[5] Gandhi S., Abramov A.Y. (2012). Mechanism of oxidative stress in neurodegeneration. Oxid. Med. Cell. Longev..

[6] Rochette L., Lorin J., Zeller M., Guilland J.C., Lorgis L., Cottin Y., Vergely C. (2013). Nitric oxide synthase inhibition and oxidative stress in cardiovascular diseases: Possible therapeutic targets?. Pharmacol. Ther..

[7] Dozor A.J. (2010). The role of oxidative stress in the pathogenesis and treatment of asthma. Ann. N. Y. Acad. Sci..

[8] Zhou R., Tardivel A., Thorens B., Choi I., Tschopp J. (2010). Thioredoxin-interacting protein links oxidative stress to inflammasome activation. Nat. Immunol..

[9] Hitchon C.A., El-Gabalawy H.S. (2004). Oxidation in rheumatoid arthritis. Arthritis Res. Ther..

[10] Griffiths H.R. (2005). ROS as signalling molecules in T cells–evidence for abnormal redox signalling in the autoimmune disease, rheumatoid arthritis. Redox Rep..

[11] Schapira A.H. (2008). Mitochondria in the aetiology and pathogenesis of Parkinson’s disease. Lancet Neurol..

[12] Keaney J.F., Larson M.G., Vasan R.S., Wilson P.W., Lipinska I., Corey D., Massaro J.M., Sutherland P., Vita J.A., Benjamin E.J. (2003). Obesity and systemic oxidative stress: Clinical correlates of oxidative stress in the Framingham Study. Arterioscler. Thromb. Vasc. Biol..

[13] Rao F., Zhang K., Khandrika S., Mahata M., Fung M.M., Ziegler M.G., Rana B.K., O’Connor D.T. (2010). Isoprostane, an “Intermediate Phenotype” for Oxidative Stress: Heritability, Risk Trait Associations, and the Influence of Chromogranin B Polymorphism. J. Am. Coll. Cardiol..

[14] Emelyanov A., Fedoseev G., Abulimity A., Rudinski K., Fedoulov A., Karabanov A., Barnes P.J. (2001). Elevated concentrations of exhaled hydrogen peroxide in asthmatic patients. CHEST J..

[15] Ichinose M., Sugiura H., Yamagata S., Koarai A., Shirato K. (2000). Increase in reactive nitrogen species production in chronic obstructive pulmonary disease airways. Am. J. Respir. Crit. Care.

[16] Ganas K., Loukides S., Papatheodorou G., Panagou P., Kalogeropoulos N. (2001). Total nitrite/nitrate in expired breath condensate of patients with asthma. Respir. Med..

[17] Kharitonov S.A., Yates D., Springall D.R., Buttery L., Polak J., Robbins R.A., Barnes P.J. (1995). Exhaled nitric oxide is increased in asthma. CHEST.

[18] Maritim A.C., Sanders A., Watkins J. (2003). Diabetes, oxidative stress, and antioxidants: A review. J. Biochem. Mol. Toxicol..

[19] Jami M.S., Hemati S., Salehi Z., Tavassoli M. (2008). Association between the length of a CA dinucleotide repeat in the EGFR and risk of breast cancer. Cancer Investig..

[20] Ahmadinejad F., Honardoost M.A., Mowla S.J., Teimori H., Ghaedi K. (2016). miR-218 as a Multifunctional Regulator of Oncogenic Processes in Different Solid Tumors. Genet. 3rd Millenn..

[21] Mahmoudian-sani M.R., Mehri-Ghahfarrokhi A., Ahmadinejad F., Hashemzadeh-Chaleshtori M., Saidijam M., Jami M.S. (2017). MicroRNAs: Effective elements in ear-related diseases and hearing loss. Eur. Arch. Otorhinolaryngol..

[22] Hashemzadeh-Chaleshtori M., Saidijam M., Jami M.S., Ghasemi-Dehkordi P. (2016). MicroRNA-183 Family in Inner Ear: Hair Cell Development and Deafness. J. Audiol. Otol..

[23] Evans M.D., Dizdaroglu M., Cooke M.S. (2004). Oxidative DNA damage and disease: Induction, repair and significance. Mutat. Res.-Rev. Mutat..

[24] Jami M.S., Hou J., Liu M., Varney M.L., Hassan H., Dong J., Geng L., Wang J., Yu F., Huang X. (2014). Functional proteomic analysis reveals the involvement of KIAA1199 in breast cancer growth, motility and invasiveness. BMC Cancer.

[25] García-Estrada C., Barreiro C., Jami M.-S., Martín-González J., Martín J.-F. (2013). The inducers 1,3-diaminopropane and spermidine cause the reprogramming of metabolism in *Penicillium chrysogenum*, leading to multiple vesicles and penicillin overproduction. J. Proteomics.

[26] Jami M.S., García-Estrada C., Barreiro C., Cuadrado A.A., Salehi-Najafabadi Z., Martín J.F. (2010). The Penicillium chrysogenum extracellular proteome. Conversion from a food-rotting strain to a versatile cell factory for white biotechnology. Mol. Cell. Proteomics.

[27] Li C.L., Werner P., Cohen G. (1995). Lipid peroxidation in brain: Interactions of L-DOPA/dopamine with ascorbate and iron. Neurodegeneration.

[28] Reitz C., Brayne C., Mayeux R. (2011). Epidemiology of Alzheimer disease. Nat. Rev. Neurol..

[29] Harkany T., Penke B., Luiten P.G. (2000). β-Amyloid Excitotoxicity in Rat Magnocellular Nucleus Basalis: Effect of Cortical Deafferentation on Cerebral Blood Flow Regulation and Implications for Alzheimer's Disease. Ann. N. Y. Acad. Sci..

[30] Greig N.H., Mattson M.P., Perry T., Chan S.L., Giordano T., Sambamurti K., Rogers J.T., Ovadia H., Lahiri D.K. (2004). New Therapeutic Strategies and Drug Candidates for Neurodegenerative Diseases: p53 and TNF-α Inhibitors, and GLP-1 Receptor Agonists. Ann. N. Y. Acad. Sci..

[31] Andreyev A.Y., Kushnareva Y.E., Starkov A. (2005). Mitochondrial metabolism of reactive oxygen species. Biochemistry (Moscow).

[32] Keating D.J. (2008). Mitochondrial dysfunction, oxidative stress, regulation of exocytosis and their relevance to neurodegenerative diseases. J. Neurochem..

[33] Dalfó E., Portero-Otín M., Ayala V., Martínez A., Pamplona R., Ferrer I. (2005). Evidence of oxidative stress in the neocortex in incidental Lewy body disease. J. Neuropathol. Exp. Neurol..

[34] Heisters D. (2011). Parkinson’s: Symptoms, treatments and research. Br. J. Nurs..

[35] Jankovic J. (2008). Parkinson’s disease: Clinical features and diagnosis. J. Neurol. Neurosurg. Psychiatry.

[36] Polymeropoulos M.H., Lavedan C., Leroy E., Ide S.E., Dehejia A., Dutra A., Pike B., Root H., Rubenstein J., Boyer R. (1997). Mutation in the α-synuclein gene identified in families with Parkinson’s disease. Science.

[37] Gilks W.P., Abou-Sleiman P.M., Gandhi S., Jain S., Singleton A., Lees A.J., Shaw K., Bhatia K.P., Bonifati V., Quinn N.P. (2005). A common LRRK2 mutation in idiopathic Parkinson’s disease. Lancet.

[38] Bonifati V., Rizzu P., Van Baren M.J., Schaap O., Breedveld G.J., Krieger E., Dekker M.C., Squitieri F., Ibanez P., Joosse M. (2003). Mutations in the DJ-1 gene associated with autosomal recessive early-onset parkinsonism. Science.

[39] Valente E.M., Abou-Sleiman P.M., Caputo V., Muqit M.M., Harvey K., Gispert S., Ali Z., Del Turco D., Bentivoglio A.R., Healy D.G. (2004). Hereditary early-onset Parkinson’s disease caused by mutations in PINK1. Science.

[40] Kitada T., Asakawa S., Hattori N., Matsumine H., Yamamura Y., Minoshima S., Yokochi M., Mizuno Y., Shimizu N. (1998). Mutations in the parkin gene cause autosomal recessive juvenile parkinsonism. Nature.

[41] D’Amelio M., Ragonese P., Sconzo G., Aridon P., Savettieri G. (2009). Parkinson’s disease and cancer: Insights for pathogenesis from epidemiology. Ann. N. Y. Acad. Sci..

[42] Jenner P. (1996). Oxidative stress in Parkinson’s disease and other neurodegenerative disorders. Pathol. Biol..

[43] Sian J., Dexter D.T., Lees A.J., Daniel S., Agid Y., Javoy-Agid F., Jenner P., Marsden C.D. (1994). Alterations in glutathione levels in Parkinson’s disease and other neurodegenerative disorders affecting basal ganglia. Ann. Neurol..

[44] Jellinger K.A., Kienzl E., Rumpelmaier G., Paulus W., Riederer P., Stachelberger H., Youdim M.B., Ben-Shachar D. (1993). Iron and ferritin in substantia nigra in Parkinson’s disease. Adv. Neurol..

[45] Cadet J., Douki T., Ravanat J.-L. (2010). Oxidatively generated base damage to cellular DNA. Free Radic. Biol. Med..

[46] Altieri F., Grillo C., Maceroni M., Chichiarelli S. (2008). DNA damage and repair: From molecular mechanisms to health implications. Antioxid. Redox Sign..

[47] Banerjee B., Seth V., Bhattacharya A., Pasha S., Chakraborty A. (1999). Biochemical effects of some pesticides on lipid peroxidation and free-radical scavengers. Toxicol. Lett..

[48] Chalansonnet M., Carabin N., Boucard S., Cosnier F., Nunge H., Gagnaire F. (2013). Study of the potential oxidative stress induced by six solvents in the rat brain. Neurotoxicology.

[49] Jomova K., Valko M. (2011). Advances in metal-induced oxidative stress and human disease. Toxicology.

[50] De Bont R., Van Larebeke N. (2004). Endogenous DNA damage in humans: A review of quantitative data. Mutagenesis.

[51] Ghasemi-Dehkordi P., Allahbakhshian-Farsani M., Abdian N., Mirzaeian A., Saffari-Chaleshtori J., Heybati F., Mardani G., Karimi-Taghanaki A., Doosti A., Jami M.S. (2015). Comparison between the cultures of human induced pluripotent stem cells (hiPSCs) on feeder-and serum-free system (Matrigel matrix), MEF and HDF feeder cell lines. J. Cell Commun. Sign..

[52] Cairns R.A., Harris I.S., Mak T.W. (2011). Regulation of cancer cell metabolism. Nat. Rev. Cancer.

[53] Sena L.A., Chandel N.S. (2012). Physiological roles of mitochondrial reactive oxygen species. Mol. Cell.

[54] Valko M., Rhodes C., Moncol J., Izakovic M., Mazur M. (2006). Free radicals, metals and antioxidants in oxidative stress-induced cancer. Chem. Biol. Interact..

[55] Poli G., Leonarduzzi G., Biasi F., Chiarpotto E. (2004). Oxidative stress and cell signalling. Curr. Med. Chem..

[56] Decoursey T., Ligeti E. (2005). Regulation and termination of NADPH oxidase activity. Cell. Mol. Life Sci..

[57] Hsu T.C., Young M.R., Cmarik J., Colburn N.H. (2000). Activator protein 1 (AP-1)–and nuclear factor κB (NF-κB)–dependent transcriptional events in carcinogenesis. Free Rad. Biol. Med..

[58] Rhee S.G. (2006). H_2_O_2_, a necessary evil for cell signaling. Science.

[59] Rosen H., Klebanoff S.J., Wang Y., Brot N., Heinecke J.W., Fu X. (2009). Methionine oxidation contributes to bacterial killing by the myeloperoxidase system of neutrophils. Proc. Natl. Acad. Sci. USA.

[60] Bashan N., Kovsan J., Kachko I., Ovadia H., Rudich A. (2009). Positive and negative regulation of insulin signaling by reactive oxygen and nitrogen species. Physiol. Rev..

[61] Thannickal V.J., Fanburg B.L. (2000). Reactive oxygen species in cell signaling. Am. J. Physiol. Lung Cell. Mol. Physiol..

[62] Schwab L., Goroncy L., Palaniyandi S., Gautam S., Triantafyllopoulou A., Mocsai A., Reichardt W., Karlsson F.J., Radhakrishnan S.V., Hanke K. (2014). Neutrophil granulocytes recruited upon translocation of intestinal bacteria enhance graft-versus-host disease via tissue damage. Nat. Med..

[63] Kiley P.J., Storz G. (2004). Exploiting thiol modifications. PLoS Biol..

[64] Reddie K.G., Carroll K.S. (2008). Expanding the functional diversity of proteins through cysteine oxidation. Curr. Opin. Chem. Biol..

[65] Butterfield D.A. (2004). Proteomics: A new approach to investigate oxidative stress in Alzheimer’s disease brain. Brain Res..

[66] Castegna A., Aksenov M., Thongboonkerd V., Klein J.B., Pierce W.M., Booze R., Markesbery W.R., Butterfield D.A. (2002). Proteomic identification of oxidatively modified proteins in Alzheimer’s disease brain. Part II: Dihydropyrimidinase-related protein 2, α-enolase and heat shock cognate 71. J. Neurochem..

[67] Castegna A., Thongboonkerd V., Klein J.B., Lynn B., Markesbery W.R., Butterfield D.A. (2003). Proteomic identification of nitrated proteins in Alzheimer’s disease brain. J. Neurochem..

[68] Basso M., Giraudo S., Corpillo D., Bergamasco B., Lopiano L., Fasano M. (2004). Proteome analysis of human substantia nigra in Parkinson’s disease. Proteomics.

[69] Castegna A., Aksenov M., Aksenova M., Thongboonkerd V., Klein J.B., Pierce W.M., Booze R., Markesbery W.R., Butterfield D.A. (2002). Proteomic identification of oxidatively modified proteins in Alzheimer’s disease brain. Part I: Creatine kinase BB, glutamine synthase, and ubiquitin carboxy-terminal hydrolase L-1. Free Radic. Biol. Med..

[70] Mattson M.P., Gary D.S., Chan S.L., Duan W. (2001). Perturbed endoplasmic reticulum function, synaptic apoptosis and the pathogenesis of Alzheimer’s disease. Biochem. Soc. Symp..

[71] Glaser C.B., Yamin G., Uversky V.N., Fink A.L. (2005). Methionine oxidation, α-synuclein and Parkinson’s disease. BBA-Proteins Proteo..

[72] Nordberg J., Arner E.S. (2001). Reactive oxygen species, antioxidants, and the mammalian thioredoxin system. Free Radic. Biol. Med..

[73] Chelikani P., Fita I., Loewen P.C. (2004). Diversity of structures and properties among catalases. Cell. Mol. Life Sci..

[74] Meister A., Anderson M.E. (1983). Glutathione. Ann. Rev. Biochem..

[75] Brigelius-Flohé R. (1999). Tissue-specific functions of individual glutathione peroxidases. Free Rad. Biol. Med..

[76] Arnér E.S., Holmgren A. (2000). Physiological functions of thioredoxin and thioredoxin reductase. Eur. J. Biochem..

[77] Bannister J.V., Bannister W.H., Rotilio G. (1987). Aspects of the structure, function, and applications of superoxide dismutas. CRC Crit. Rev. Biochem..

[78] Zelko I.N., Mariani T.J., Folz R.J. (2002). Superoxide dismutase multigene family: A comparison of the CuZn-SOD (SOD1), Mn-SOD (SOD2), and EC-SOD (SOD3) gene structures, evolution, and expression. Free Rad. Biol. Med..

[79] Rice M.E. (2000). Ascorbate regulation and its neuroprotective role in the brain. Trends Neurosci..

[80] Halliwell B. (2001). Role of free radicals in the neurodegenerative diseases. Drug. Aging.

[81] McCay P.B. (1985). Vitamin E: Interactions with free radicals and ascorbate. Ann. Rev. Nutr..

[82] Gilgun-Sherki Y., Melamed E., Offen D. (2001). Oxidative stress induced-neurodegenerative diseases: The need for antioxidants that penetrate the blood brain barrier. Neuropharmacology.

[83] Tan D.X., Chen L., Poeggeler B., Manchester L., Reiter R. (1993). Melatonin: A potent, endogenous hydroxyl radical scavenger. Endocr. J..

[84] Reiter R.J., Calvo J.R., Karbownik M., Qi W., Tan D.X. (2000). Melatonin and its relation to the immune system and inflammation. Ann. N. Y. Acad. Sci..

[85] Reiter R.J., Tan D.X., Acuna-Castroviejo D., Burkhardt S., Karbownik M. (2000). Melatonin: Mechanisms and actions as an antioxidant. Curr. Top. Biophys..

[86] Tan D.X., Manchester L.C., Reiter R.J., Qi W.B., Karbownik M., Calvo J.R. (2000). Significance of melatonin in antioxidative defense system: Reactions and products. Neurosignals.

[87] Turunen M., Olsson J., Dallner G. (2004). Metabolism and function of coenzyme Q. BBA-Biomembranes.

[88] Hornykiewicz O. (1998). Biochemical aspects of Parkinson’s disease. Neurology.

[89] LeWitt P.A. (2008). Levodopa for the treatment of Parkinson’s disease. N. Engl. J. Med..

[90] Mena M.A., Casarejos M.J., Solano R.M., de Yebenes J.G. (2009). Half a century of L-DOPA. Curr. Top. Med. Chem..

[91] Group EAIS (2003). Effect of a novel free radical scavenger, edaravone (MCI-186), on acute brain infarction. Randomized, placebo-controlled, double-blind study at multicenters. Cerebrovas. Dis. (Basel, Switzerland).

[92] Amemiya S., Kamiya T., Nito C., Inaba T., Kato K., Ueda M., Shimazaki K., Katayama Y. (2005). Anti-apoptotic and neuroprotective effects of edaravone following transient focal ischemia in rats. Eur. J. Pharmacol..

[93] Toyoda K., Fujii K., Kamouchi M., Nakane H., Arihiro S., Okada Y., Ibayashi S., Iida M. (2004). Free radical scavenger, edaravone, in stroke with internal carotid artery occlusion. J. Neurol. Sci..

[94] Abe K., Yuki S., Kogure K. (1988). Strong attenuation of ischemic and postischemic brain edema in rats by a novel free radical scavenger. Stroke.

[95] Yamamoto T., Yuki S., Watanabe T., Mitsuka M., Saito K.I., Kogure K. (1997). Delayed neuronal death prevented by inhibition of increased hydroxyl radical formation in a transient cerebral ischemia. Brain Res..

[96] Mercuri N.B., Bernardi G. (2005). The ‘magic’ of L-dopa: Why is it the gold standard Parkinson’s disease therapy?. Trends Pharmacol. Sci..

[97] Eiden L.E., Schäfer M.K.H., Weihe E., Schütz B. (2004). The vesicular amine transporter family (SLC18): Amine/proton antiporters required for vesicular accumulation and regulated exocytotic secretion of monoamines and acetylcholine. Pflügers Archiv..

[98] Lappin J. (2011). Book Review: The Myth of the Chemical Cure: A Critique of Psychiatric Drug Treatment. Int. J. Soc. Psychiatry.

[99] Volkow N.D., Wang G.J., Kollins S.H., Wigal T.L., Newcorn J.H., Telang F., Fowler J.S., Zhu W., Logan J., Ma Y. (2009). Evaluating dopamine reward pathway in ADHD: Clinical implications. JAMA.

[100] Blessing H., Bareiss M., Zettlmeisl H., Schwarz J., Storch A. (2003). Catechol-O-methyltransferase inhibition protects against 3, 4-dihydroxyphenylalanine (DOPA) toxicity in primary mesencephalic cultures: New insights into levodopa toxicity. Neurochem. Int..

[101] Reksidler A.B., Lima M.M., Dombrowski P.A., Barnabé G.F., Andersen M.L., Tufik S., Vital M.A. (2009). Distinct effects of intranigral L-DOPA infusion in the MPTP rat model of Parkinson’s disease. Birth, Life and Death of Dopaminergic Neurons in the Substantia Nigra.

[102] Spencer J.P., Jenner A., Butler J., Aruoma O.I., Dexter D.T., Jenner P., Halliwell B. (1996). Evaluation of the pro-oxidant and antioxidant actions of L-DOPA and dopamine in vitro: Implications for Parkinson’s disease. Free Radic. Res..

[103] Han S.K., Mytilineou C., Cohen G. (1996). L-DOPA Up-Regulates Glutathione and Protects Mesencephalic Cultures Against Oxidative Stress. J. Neurochem..

[104] Jami M.S., Pal R., Hoedt E., Neubert T.A., Larsen J.P., Møller S.G. (2014). Proteome analysis reveals roles of L-DOPA in response to oxidative stress in neurons. BMC Neurosci..

[105] Alberio T., Pippione A.C., Comi C., Olgiati S., Cecconi D., Zibetti M., Lopiano L., Fasano M. (2012). Dopaminergic therapies modulate the T-CELL proteome of patients with Parkinson’s disease. IUBMB Life.

[106] Mila S., Albo A.G., Corpillo D., Giraudo S., Zibetti M., Bucci E.M., Lopiano L., Fasano M. (2009). Lymphocyte proteomics of Parkinson’s disease patients reveals cytoskeletal protein dysregulation and oxidative stress. Biomakers.

[107] Campanella C., Bucchieri F., Ardizzone N.M., Gammazza A.M., Montalbano A., Ribbene A., Di Felice V., Bellafiore M., David S., Rappa F. (2008). Upon oxidative stress, the antiapoptotic Hsp60/procaspase-3 complex persists in mucoepidermoid carcinoma cells. Eur. J. Histochem..

[108] Takada M., Otaka M., Takahashi T., Izumi Y., Tamaki K., Shibuya T., Sakamoto N., Osada T., Yamamoto S., Ishida R. (2010). Overexpression of a 60-kDa heat shock protein enhances cytoprotective function of small intestinal epithelial cells. Life Sci..

[109] Ozawa K., Kuwabara K., Tamatani M., Takatsuji K., Tsukamoto Y., Kaneda S., Yanagi H., Stern D.M., Eguchi Y., Tsujimoto Y. (1999). 150-kDa oxygen-regulated protein (ORP150) suppresses hypoxia-induced apoptotic cell death. J. Biol. Chem..

[110] Watanabe T., Yuki S., Egawa M., Nishi H. (1994). Protective effects of MCI-186 on cerebral ischemia: Possible involvement of free radical scavenging and antioxidant actions. J. Pharmacol. Exp. Ther..

[111] Yamamoto Y., Kuwahara T., Watanabe K., Watanabe K. (1996). Antioxidant activity of 3-methyl-1-phenyl-2-pyrazolin-5-one. Redox Rep..

[112] Yuan W.J., Yasuhara T., Shingo T., Muraoka K., Agari T., Kameda M., Uozumi T., Tajiri N., Morimoto T., Jing M. (2008). Neuroprotective effects of edaravone-administration on 6-OHDA-treated dopaminergic neurons. BMC Neurosci..

[113] Jiao S.S., Yao X.Q., Liu Y.H., Wang Q.H., Zeng F., Lu J.J., Liu J., Zhu C., Shen L.L., Liu C.H. (2015). Edaravone alleviates Alzheimer’s disease-type pathologies and cognitive deficits. Proc. Nat. Acad. Sci..

[114] Xi H., Akishita M., Nagai K., Yu W., Hasegawa H., Eto M., Kozaki K., Toba K. (2007). Potent free radical scavenger, edaravone, suppresses oxidative stress-induced endothelial damage and early atherosclerosis. Atherosclerosis.

[115] Higashi Y., Jitsuiki D., Chayama K., Yoshizumi M. (2006). Edaravone (3-methyl-1-phenyl-2-pyrazolin-5-one), a novel free radical scavenger, for treatment of cardiovascular diseases. Recent Pat. Cardiovasc. Drug Discov..

[116] Hayashi T., Mori T., Sohmiya K., Okada Y., Inamoto S., Okuda N., Mori H., Kitaura Y. (2003). Efficacy of edaravone, a free radical scavenger, on left ventricular function and structure in diabetes mellitus. J. Cardiovas. Pharmacol..

[117] Onodera H., Arito M., Sato T., Ito H., Hashimoto T., Tanaka Y., Kurokawa M.S., Okamoto K., Suematsu N., Kato T. (2013). Novel effects of edaravone on human brain microvascular endothelial cells revealed by a proteomic approach. Brain Res..

[118] Jami M.S., Salehi-Najafabadi Z., Ahmadinejad F., Hoedt E., Chaleshtori M.H., Ghatrehsamani M., Neubert T.A., Larsen J.P., Møller S.G. (2015). Edaravone leads to proteome changes indicative of neuronal cell protection in response to oxidative stress. Neurochem. Int..

[119] Hu X., Weng Z., Chu C.T., Zhang L., Cao G., Gao Y., Signore A., Zhu J., Hastings T., Greenamyre J.T. (2011). Peroxiredoxin-2 protects against 6-hydroxydopamine-induced dopaminergic neurodegeneration via attenuation of the apoptosis signal-regulating kinase (ASK1) signaling cascade. J. Neurosci..

[120] Agarwal A.R., Zhao L., Sancheti H., Sundar I.K., Rahman I., Cadenas E. (2012). Short-term cigarette smoke exposure induces reversible changes in energy metabolism and cellular redox status independent of inflammatory responses in mouse lungs. Am. J. Physiol. Lung Cell. Mol. Physiol..

[121] Ralser M., Wamelink M.M., Kowald A., Gerisch B., Heeren G., Struys E.A., Klipp E., Jakobs C., Breitenbach M., Lehrach H. (2007). Dynamic rerouting of the carbohydrate flux is key to counteracting oxidative stress. J. Biol..

[122] Varticovski L., Chahwala S.B., Whitman M., Cantley L., Schindler D., Chow E.P., Sinclair L.K., Pepinsky R.B. (1988). Location of sites in human lipocortin I that are phosphorylated by protein tyrosine kinases and protein kinases A and C. Biochemistry.

[123] Zhang Z., Huang L., Zhao W., Rigas B. (2010). Annexin 1 induced by anti-inflammatory drugs binds to NF-κB and inhibits its activation: Anticancer effects in vitro and in vivo. Cancer Res..

[124] Fisher A.B. (2011). Peroxiredoxin 6: A bifunctional enzyme with glutathione peroxidase and phospholipase A2 activities. Antioxid. Redox Signal..

[125] Tulsawani R., Kelly L.S., Fatma N., Chhunchha B., Kubo E., Kumar A., Singh D.P. (2010). Neuroprotective effect of peroxiredoxin 6 against hypoxia-induced retinal ganglion cell damage. BMC Neurosci..

[126] Ariga H., Takahashi-Niki K., Kato I., Maita H., Niki T., Iguchi-Ariga S.M. (2013). Neuroprotective function of DJ-1 in Parkinson’s disease. Oxid. Med. Cell. Longev..

[127] Fan J., Ren H., Jia N., Fei E., Zhou T., Jiang P., Wu M., Wang G. (2008). DJ-1 decreases Bax expression through repressing p53 transcriptional activity. J. Biol. Chem..

[128] Kos J., Jevnikar Z., Obermajer N. (2009). The role of cathepsin X in cell signaling. Cell Adhes. Migr..

[129] Bravo-Cordero J.J., Magalhaes M.A., Eddy R.J., Hodgson L., Condeelis J. (2013). Functions of cofilin in cell locomotion and invasion. Nat. Rev. Mol. Cell Biol..

[130] Lin J.J.C., Eppinga R.D., Warren K.S., McCrae K.R. (2008). Human tropomyosin isoforms in the regulation of cytoskeleton functions. Tropomyosin.

